# Microwave Sensor for Dielectric Constant of Lossy Organic Liquids Based on Negative-Resistance Oscillation

**DOI:** 10.3390/s25030961

**Published:** 2025-02-05

**Authors:** Huan Liu, Yichao Meng

**Affiliations:** 1 Institute of Fiber Optics, Shanghai University, Shanghai 201800, China; lz335829@163.com; 2 Key Laboratory of Specialty Fiber Optics and Optical Access Networks, Joint International Research Laboratory of Specialty Fiber Optics and Advanced Communication, Shanghai University, Shanghai 20044, China

**Keywords:** permittivity measurement, oscillator, dielectric constant, sensor, liquids

## Abstract

The dielectric constant, or permittivity, is a fundamental property that characterizes a material’s electromagnetic behavior, crucial for diverse applications in agriculture, healthcare, industry, and scientific research. In microwave engineering, accurate permittivity measurement is essential for advancements in fields such as biomedicine, aerospace, and microwave chemistry. However, conventional waveguide resonator methods face challenges when measuring high-loss materials, often leading to reduced accuracy and increased cost. This paper introduces a lightweight, compact system for dielectric constant measurement using a negative-resistance voltage-controlled oscillator (VCO) integrated within a frequency synthesizer. The proposed system employs phase response variations of a planar sensor embedded in the VCO’s gate network to detect changes in oscillation frequency, enabling precise measurement of high-loss materials. The experimental validation demonstrates the system’s capability to accurately measure dielectric constants of lossy organic liquids, with applications in distinguishing liquid mixtures. The contributions include the design of a resonant-network-attached oscillator, comprehensive sensor performance simulations, and successful characterization of organic liquid mixtures, showcasing the potential of this approach for practical dielectric property measurements.

## 1. Introduction

The dielectric constant (aka permittivity) is a fundamental property that characterizes a material’s electromagnetic behavior [1]. It plays a critical role across diverse fields, such as agriculture [2], chemistry [3], healthcare [4], medicine [5], biology [6,7], industry [8,9], and scientific research [10,11]. In the realm of microwave engineering, accurate measurement of permittivity is paramount for advancing technologies and applications in areas such as microwave chemistry, aerospace, biomedicine, and materials science [12]. This growing importance is underscored by extensive research efforts aimed at characterizing the dielectric properties of human tissues, body fluids, and cells, which are crucial for disease diagnosis and treatment [13,14].

Conventional microwave techniques often utilize waveguide resonators to determine permittivity through changes in resonant frequency and quality factor (i.e., *Q*-factor). However, these methods face significant challenges when applied to high-loss materials such as ethanol, methanol, high-loss wine [15], and other liquids [16]. The pronounced degradation of the resonator’s *Q*-factor in such cases diminishes the distinct peaks or notches in *S*-parameter responses, rendering accurate permittivity measurements difficult and costly [17]. Efforts to mitigate this, such as reducing the sample volume of the material under test (MUT) to lower resonator loading, can inadvertently complicate measurements by diminishing frequency shifts caused by the MUT [18]. A particular study [19] introduces valuable guidance for the fabrication of flexible materials with negative permittivity and addresses the issue of high-frequency dispersion associated with negative permittivity. Moreover, modern microwave circuit designs emphasize the miniaturization of components to eliminate reliance on bulky laboratory equipment. This necessity has spurred interest in advanced resonator technologies.

Dielectric resonator oscillators (DROs) have emerged as a promising solution due to their integration capabilities, compactness, cost-effectiveness, reliability, and stability. Widely adopted in civilian and military applications, DROs exist primarily in two forms: series-reflective and parallel-feedback designs [20]. The series-reflective DRO offers advantages such as superior phase noise and easier modeling but encounters tuning challenges due to its unfixed dielectric resonator (DR) position. Conversely, the parallel-feedback DRO utilizes the DR as a frequency-selective feedback network, offering precise frequency control and enhanced stability.

In this paper, we propose a lightweight system for measuring the dielectric constant, leveraging a negative-resistance voltage-controlled oscillator (VCO) [20] integrated within an existing frequency synthesizer. The system exploits the phase response variation of a planar sensor embedded in the VCO’s gate network and the corresponding changes in oscillation frequency to detect dielectric constants. By utilizing the phase response, the system can handle lossy test sample volumes of up to 300 μL while maintaining stable oscillations. This approach enables significant frequency shifts and improved resolution in dielectric constant measurement [21]. The experimental results demonstrate the system’s ability to measure dielectric constants with sufficient accuracy to distinguish and quantify mixtures. Furthermore, the system’s utility is showcased through its successful detection of the composition of organic liquid mixtures [22,23]. The main contributions of this work include the following:

- Design: The development of a resonant-network-attached oscillator exhibiting both inductive and capacitive characteristics.
- Simulation: A comprehensive analysis of sensor performance across various conditions.
- Detection: The accurate identification of the composition of organic liquid mixtures using the proposed oscillator design.

## 2. Oscillators for Permittivity Measurement

**Figure 1:**
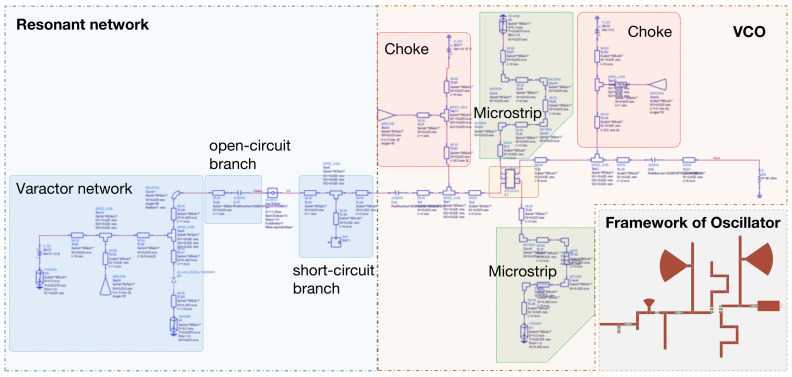
Circuit diagram of the resonant-network-attached oscillator. The framework of the oscillator is at the right bottom.

Figure 1 illustrates the resonant-network-attached negative-resistance oscillator. Its framework, highlighting the core structure, is depicted in the lower-right corner. The voltage-controlled oscillator (VCO) features two chokes and two microstrips. The resonant network consists of three primary components: the varactor network, the open-circuit branch, and the short-circuit branch.

In the circuit, the short-circuit branch exhibits inductive characteristics while the open-circuit branch exhibits capacitive characteristics. Together, they form a parallel resonant network. As this network is central to the design of the voltage-controlled oscillator (VCO), it is essential to consider the influence of the voltage-controlled varactor diode, which adjusts its capacitance based on the applied voltage. Consequently, the varactor diode network is connected in parallel with the open-circuit branch to contribute capacitance to the overall parallel resonant network. According to the parallel resonance formula, the capacitance values of the varactor network and the open-circuit branch combine to determine the total capacitance of the network.

In the following sections, we delve into the detailed design and operation of the oscillator. We also outline the simulation and testing processes for the sensor, providing a comprehensive overview of the methods employed to evaluate its performance and accuracy.

### 2.1. Oscillator Design

**Figure 2:**
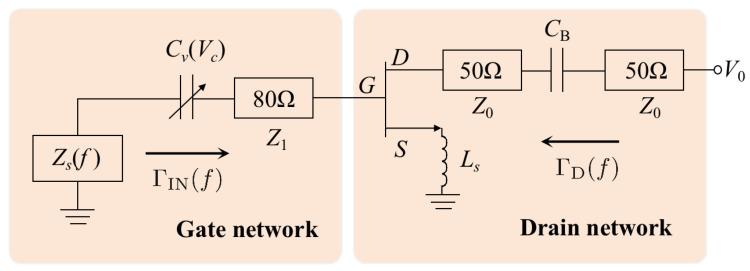
Simplified schematic of the negative-resistance oscillator used for permittivity measurement.

The oscillator comprises a drain network and a gate network, as shown in Figure 2.

The gate network incorporates a transmission line with a characteristic impedance of $Z_{1}=80\;\;\Omega$, positioned beneath a voltage-controlled varactor, $C_{v}\left(V_{c}\right)$, and a sensing element with a complex impedance $Z_{s}\left(f\right)$. When a material under test (MUT) with frequency-dependent relative permittivity is introduced to the sensor, the impedance shifts as $Z_{s}\left(f\right)$, which encapsulates the dielectric constant ε(f) and the MUT’s loss factor ℓ(f).

The oscillation frequency is governed by the combined impedance and the voltage-controlled varactor, which compensates for frequency variations induced by the MUT. The resulting oscillating signal is transmitted through the drain network. This network comprises two transmission lines with characteristic impedances of $Z_{0}=50\;\;\Omega$ and arbitrary electrical lengths, separated by a DC-blocking capacitor.

The determination of $L_{s}$ is a critical aspect in designing an oscillator with an output frequency of $f_{0}$. The inductance $L_{s}$ must be carefully tuned to ensure that the transistor exhibits negative resistance at the gate, as indicated by an input reflection coefficient |Γ|>1. To achieve this, the *S*-parameters of a properly biased transistor, terminated with 50Ω loads at both the gate and drain, are simulated using its nonlinear model to determine the optimal value of $L_{s}$.

**Figure 3:**
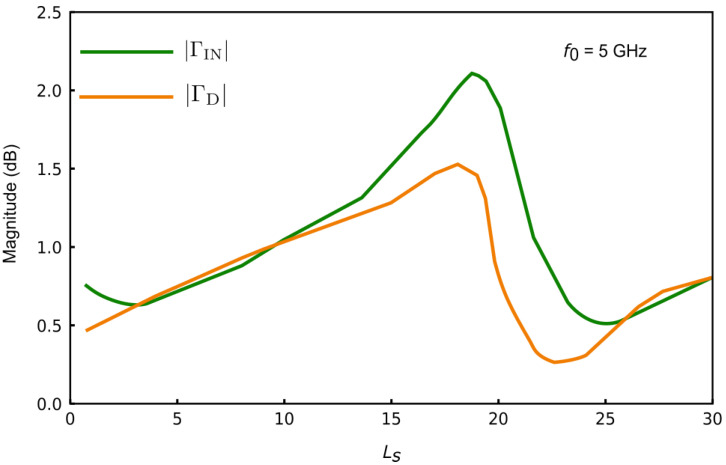
Variation in the magnitude of the reflection coefficient at the gate and drain with respect to $L_{s}$.

Figure 3 shows the variation in $\Gamma_{\mathrm{IN}}$ and $\Gamma_{D}$ at $f_{0}=5\;\mathrm{GHz}$ for different values of $L_{s}$ connected to the source terminal of the transistor. To effectively measure high-loss MUTs, the negative resistance generated must be maximized to ensure stable oscillations. The value of $L_{s}$ must be selected so that the magnitudes of the reflection coefficients at the gate and drain are maximized. Choosing $L_{s}=15$ results in $\Gamma_{\mathrm{IN}}=1.49$ and $\Gamma_{D}=1.31$, which satisfy the requirements for stable oscillation and ensure the generation of negative resistance. Since the overall network looking into the gate is capacitive, the gate network should be made inductive to fulfill the oscillation condition:

(1) $$\begin{array}{cc} ∠\Gamma_{\mathrm{IN}}\left(f_{0}\right)+∠\Gamma_{g}\left(\varepsilon ,V_{c},f_{0}\right) & =0 \end{array}$$

(2) $$\begin{array}{cc} |\Gamma_{\mathrm{IN}}\left(f_{0}\right)\left|\times \right|\Gamma_{g}\left(\varepsilon ,V_{c},f_{0}\right)| & >1 \end{array}$$

. This assures stable oscillations at a frequency $f_{0}$. Here, $\Gamma_{\mathrm{IN}}$ is the input gate reflection coefficient and $\Gamma_{g}$ is the reflection coefficient of the resonant network. The dielectric constant is given by ε and the voltage is given by $V_{c}$ [24].

**Figure 4:**
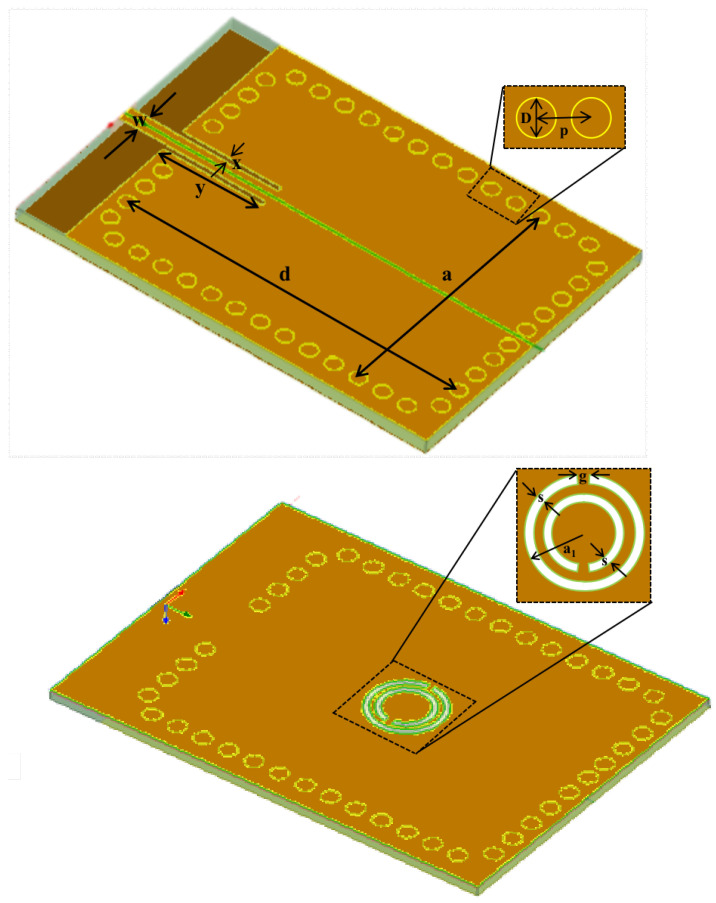
Dimensions of the SRR: (**top**) front view and (**bottom**) rear view.

In the gate network, following the approach in [20], the sensing element is a split-ring resonator (SRR) coupled to a microstrip line, as shown in Figure 4. The basic design of the proposed sensor is carried out in two steps: first, the design of the basic SIW cavity, and second, the placement of the SRR on the bottom wall of the SIW cavity. The basic SIW cavity is designed using the well-known resonance frequency expression for the TE_101_ mode [25] of operation in a rectangular SIW cavity [26,27] as follows:

(3) $$\begin{array}{cc} \; & f=\frac{1}{2\pi \sqrt{\mu \varepsilon }}\sqrt{{\left(\frac{m\pi }{a_{\mathrm{eq}}}\right)}^{2}+{\left(\frac{n\pi }{d_{\mathrm{eq}}}\right)}^{2}} \end{array}$$

(4) $$\begin{array}{cc} \; & a_{\mathrm{eq}}=a-1.08\frac{D^{2}}{p}+0.1\frac{D^{2}}{a} \end{array}$$

(5) $$\begin{array}{cc} \; & d_{\mathrm{eq}}=d-1.08\frac{D^{2}}{p}+0.1\frac{D^{2}}{d} \end{array}$$

. Here, resonance frequency *f* is dependent on the substrate permeability μ and the dielectric constant ε. *D* is the diameter of metalized vias, *p* is the center-to-center distance between two consecutive vias. The effective width $a_{\mathrm{eq}}$ of the SIW cavity is determined based on the equivalent width, *a*, of the SIW resonator and the center-to-center distance between two consecutive vias. The effective length $d_{\mathrm{eq}}$ of the SIW cavity is determined based on the equivalent length, *d*, of the SIW resonator. The constants *m* and *n* take a value of 1 in this study. Based on the above design equations, the SIW cavity is designed for a resonance frequency of 5 GHz, resonating at the fundamental TE_101_ mode. The substrate chosen is a 0.762 mm thick F4B material with a dielectric constant of ε=2.2 and a loss tangent tanδ=0.0009 (where δ represents the conductivity). The other design parameters are summarized in Table 1. After designing the SIW cavity, the SRR is etched at the center of the backside of the cavity.

### 2.2. Simulation of Sensor

**Figure 5:**
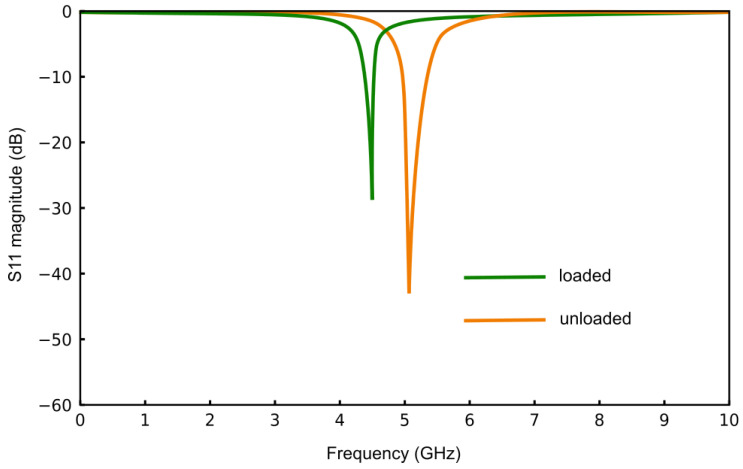
Reflection coefficient of the simulated sensor, with and without the sample placed on top of the SRR.

The SRR-loaded SIW cavity sensor is simulated using the 3D simulation software CST Studio Suite 2023 with the frequency domain solver. The simulated reflection coefficient of the SRR-loaded SIW sensor under unloaded conditions is shown in Figure 5. To determine the sensitivity of the designed sensor, a sample of size 7×7 mm_2_ with a height of 4 mm and a permittivity of 1.5 is placed over the SRR. The reflection coefficient response for this configuration is shown in Figure 5 under the label “loaded”. In this case, the resonance frequency is 5 GHz, providing a sensitivity of approximately 210 MHz for a change in permittivity of 0.5. The resonance frequency of the fabricated sensor is also measured to be 5.12 GHz, which is slightly shifted from the simulated value. This shift is due to unavoidable minor fabrication errors in the etching of the SRR. However, both the simulated and measured responses exhibit similar resonant behavior, which is the primary requirement in this context.

The unloaded quality factor ($Q_{u}$) of the substrate-integrated waveguide (SIW) resonant cavity is dependent on both the quality factor associated with the absence of dielectric losses ($Q_{c}$) and the quality factor associated with the absence of ohmic losses ($Q_{d}$). This relationship can be expressed as follows:

(6) $$\begin{array}{cc} \frac{1}{Q_{u}} & =\frac{1}{Q_{c}}+\frac{1}{Q_{d}} \end{array}$$

(7) $$\begin{array}{cc} Q_{d} & =\frac{e^{\prime }}{e^{\prime \prime }}=\frac{1}{\tan\;\delta }. \end{array}$$

In this analysis, $\epsilon^{\prime }$ and $\epsilon^{\prime \prime }$ denote the real and imaginary components of the substrate’s relative permittivity (dielectric constant), respectively. The loss tangent, tanδ, is defined as the ratio of the imaginary component ($\epsilon^{\prime \prime }$) to the real component ($\epsilon^{\prime }$) of the permittivity. This parameter is of significant importance in evaluating the performance of substrate materials in high-frequency applications.

**Figure 6:**
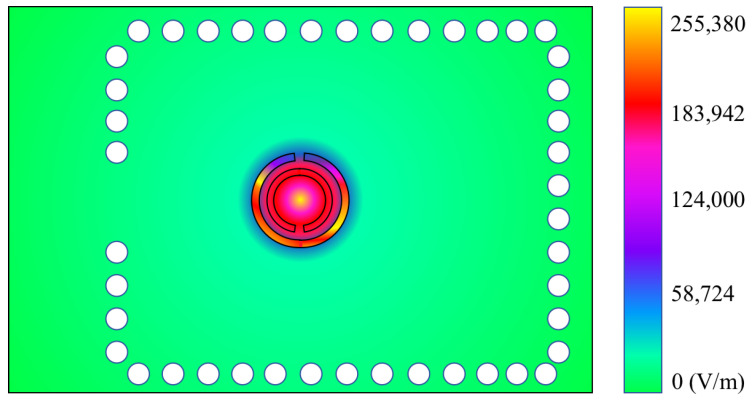
Electric field distribution on the bottom surface of unloaded sensor at the resonance.

The integration of the SRR with the SIW cavity induces a shift in the sensor’s resonance frequency to 5 GHz. This phenomenon is corroborated by the electric field distribution observed at the bottom surface of the cavity, depicted in Figure 6, which unequivocally demonstrates the excitation of the SRR. The resulting high electric field concentration in the vicinity of the SRR provides a convenient platform for direct sample placement, rendering it suitable for scanning applications.

In order to facilitate the measurement of MUTs exhibiting high losses, it is imperative to maximize the generated negative resistance to guarantee the establishment of stable oscillations. Accordingly, a comprehensive analysis of circuit stability was conducted across a range of frequencies. We use ADS momentum for our simulations.

**Figure 7:**
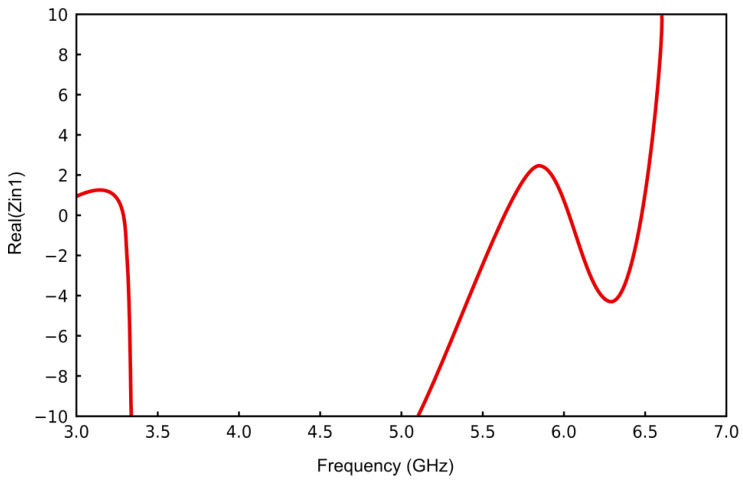
Gate input resistance.

The simulation results, illustrated in Figure 7, unequivocally demonstrate that the stability factor is less than unity within the frequency range of 3.3 GHz to 6.5 GHz, extending up to 7.0 GHz, thereby indicating a condition of instability within the circuit. Real(Zin1) denotes the real component of the input impedance measured at port 1.

**Figure 8:**
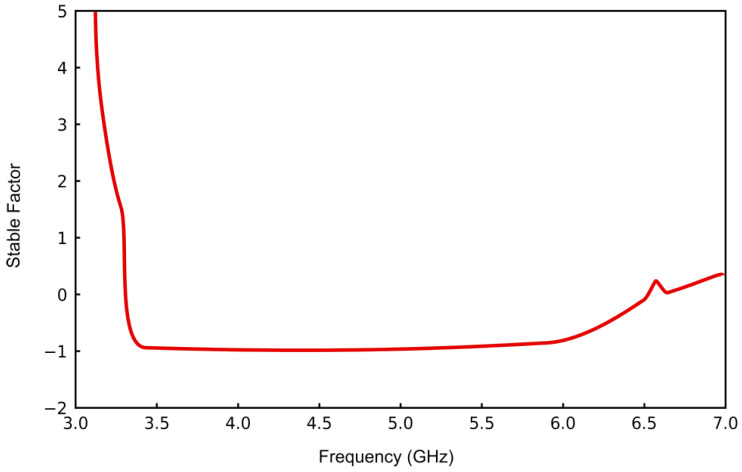
Circuit stability simulation.

The stable operation of the VCO is confirmed by the results presented in Figure 8, which indicate that the input resistance of the circuit resides in a negative-resistance state within the frequency range spanning 3.3 GHz to 6.5 GHz. This behavior is consistent with the pre-established design objectives.

**Figure 9:**
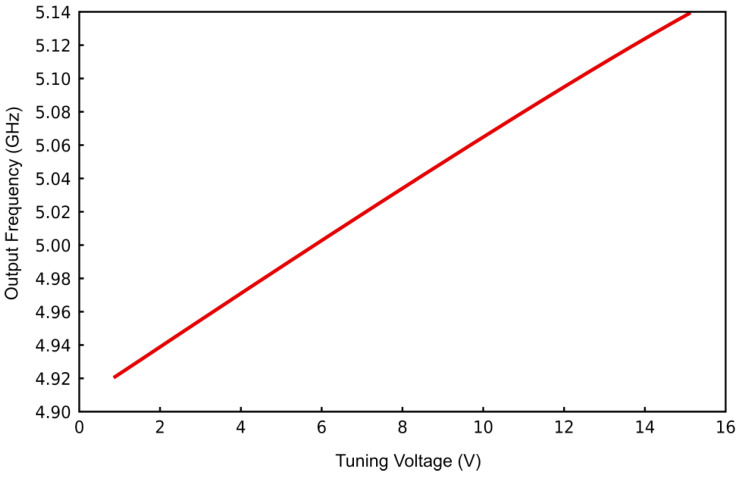
Change in output frequency ($f_{0}$) with varying voltage.

An investigation into the impact of the varactor diode bias voltage on the VCO’s operational characteristics was conducted by systematically varying the input voltage from 1 V to 15 V. The resultant tuning characteristic, illustrated in Figure 9, manifests a tuning sensitivity (slope) of 62 MHz/V and a total tuning range of 250 MHz, encompassing the frequency range from 4.915 GHz to 5.1364 GHz. These findings establish a linear dependence between the VCO output frequency and the applied bias voltage.

**Figure 10:**
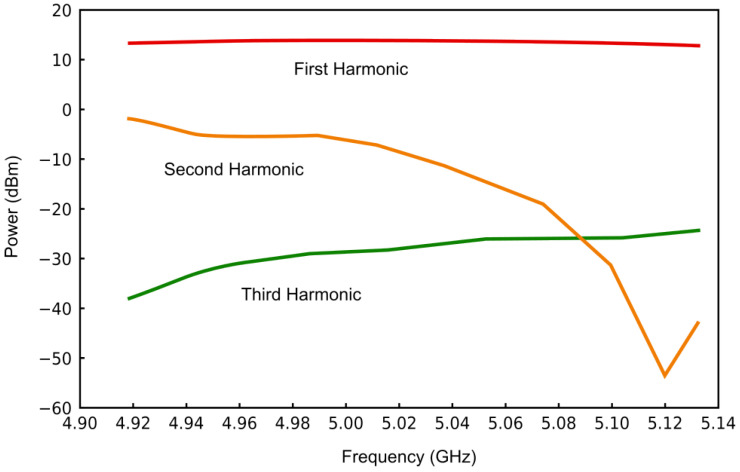
Change in resonance output power with varying frequency.

Figure 10 illustrates the output power across multiple harmonic components. The focus is on the first harmonic, as it exhibits higher and more stable output power when the frequency ranges from 4.915 GHz to 5.13 GHz. In contrast, the second harmonic produces unstable power that sharply decreases to below 0.50 dBm. While the third harmonic generates stable power, its level is considerably lower. These results highlight effective harmonic suppression.

**Figure 11:**
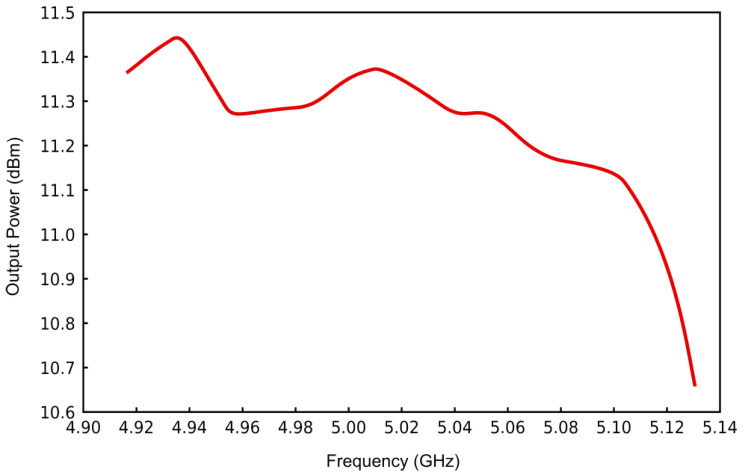
Change in VCO output power with varying frequency.

The output power characteristics of the VCO, as depicted in Figure 11, reveal that, within the frequency band spanning 4.9 GHz to 5.14 GHz, the generated output power surpasses 10.6 dBm. Concurrently, a phase noise level of −123.444 dBc is observed at a frequency offset of 100 kHz.

**Figure 12:**
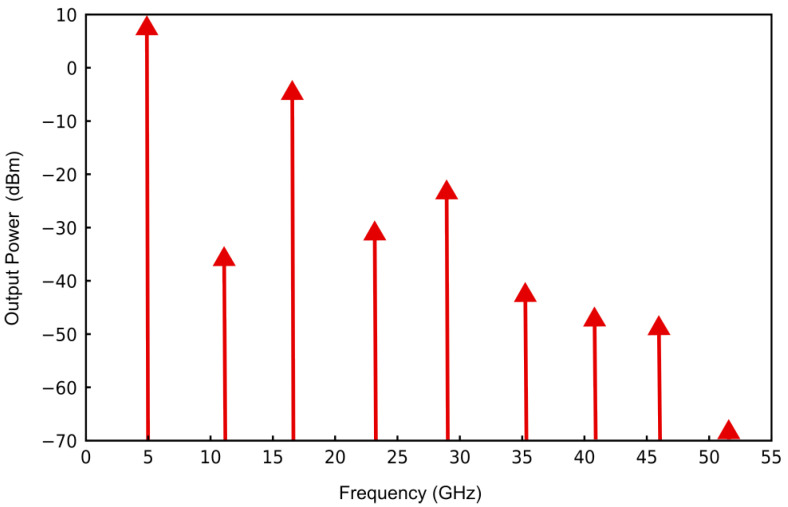
VCO output spectrum simulation.

**Figure 13:**
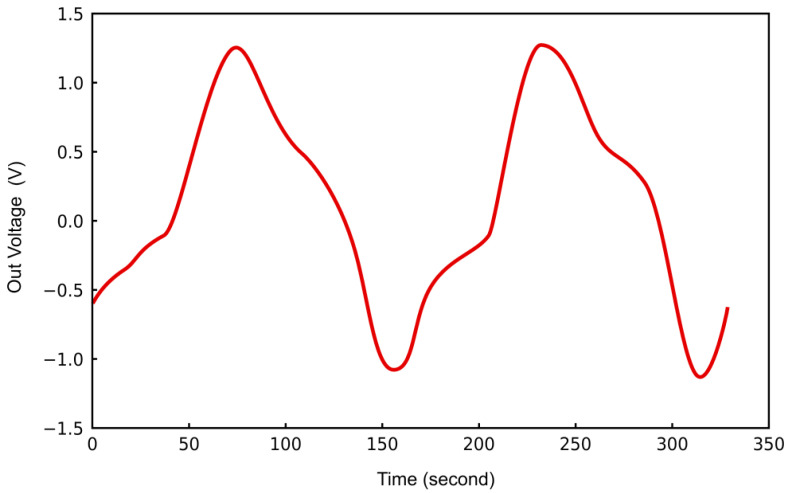
Time-domain simulation plot.

The spectral characteristics obtained from the simulation, as illustrated in Figure 12, demonstrate an output power level of 9.380 at a resonant frequency of 5 GHz, which exhibits a high degree of concordance with the results obtained from HFSS simulations. Moreover, the corresponding time-domain waveform, presented in Figure 13, demonstrates minimal waveform distortion, thereby providing strong evidence for the feasibility of the proposed design and schematic layout.

### 2.3. Implementation

**Figure 14:**
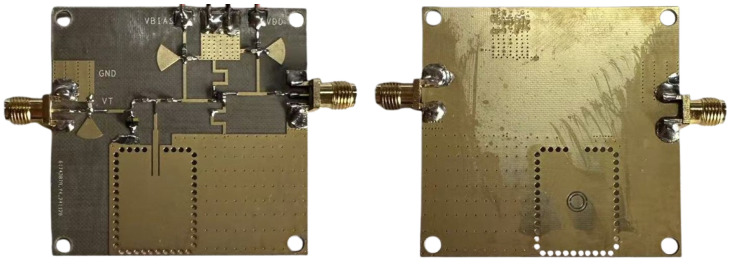
Fabricated VCO prototype: (**left**) front view and (**right**) back view.

Following numerical validation of the designed sensor’s performance utilizing simulation data, a prototype was fabricated employing a standard printed circuit board (PCB) fabrication process, with front and back views depicted in Figure 14. The requisite inductance ($L_{s}=15$ nH) was realized through the implementation of a meandered 80 Ω short-circuited transmission line stub with an electrical length of 319° to minimize the occupied substrate area. Furthermore, the microstrip transmission line comprising the sensing element was configured in an L-shaped geometry to further reduce the overall device dimensions; the parasitic effects associated with this geometric discontinuity were accounted for within the full-wave electromagnetic simulation. Figure 14 also presents the equivalent circuit representations of the varactor diode, the DC blocking capacitor, and the RF bypass capacitor.

## 3. Measurements

We began by implementing the chemical calibration and detection methodologies outlined in [20], using ethanol and methanol as reference materials due to their well-documented dielectric properties across various frequencies and temperatures [28]. The calibration process establishes a baseline characterization of the system’s response to interactions between the inhomogeneous dielectric medium under study and the electromagnetic field generated by the SRR structure.

**Figure 15:**
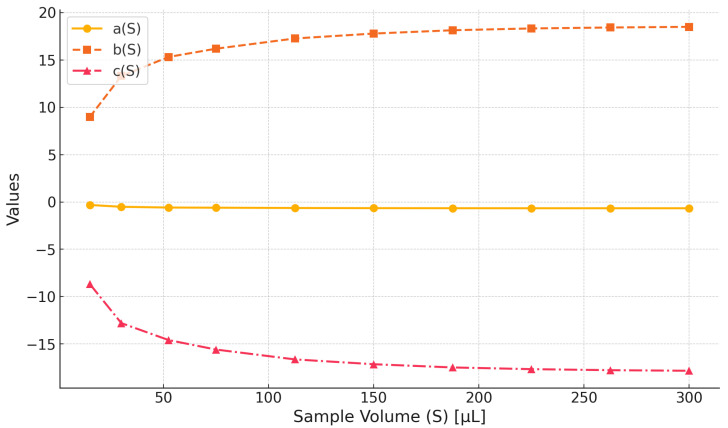
Chemical calibration coefficients.

To calculate the dielectric constant from the frequency-shift measurements for a known sample volume of the MUT, the positive roots of the polynomial introduced in [20] were computed using the calibration coefficients. Figure 15 presents the mean values of these calibration coefficients, along with their measurement uncertainties, for various sample volumes (*S*). The figure also shows the dielectric constants extracted for 2-butyl alcohol, xylene, ethyl acetate, and ethylene glycol across sample volumes ranging from 10 to 300 μL, derived from frequency shifts and the average calibration coefficients. Given that the uncertainties in the calibration coefficients are minimal, particularly for larger sample volumes, their exclusion has a negligible effect on the accuracy of detection results.

We experimentally determined the values and subjected them to a comparative analysis with theoretical predictions derived from the single relaxation time-constant Cole–Cole model [29], expressed as

(8) $$\begin{array}{c} \epsilon \left(\omega \right)=\varepsilon_{\infty }+\frac{\varepsilon_{s}-\varepsilon_{\infty }}{{\left(1+\omega^{2}t^{2}\right)}^{\left(1-\alpha \right)}}. \end{array}$$

In Equation (8), *t* is the relaxation time and α is the fitting parameter. The permittivity $\varepsilon_{s}$ is at angular frequencies $\omega \ll \frac{1}{t}$ and the permittivity $\varepsilon_{\infty }$ is at angular frequencies $\omega \gg \frac{1}{t}$.

We compare our method to [25]. The results presented in Table 2 demonstrate that our method consistently achieves lower absolute errors in permittivity measurements compared to [25], particularly for xylene and ethylene glycol, where significant improvements are observed across all measurement conditions. For 2-butyl alcohol and ethyl acetate, our method shows competitive performance, with notable reductions in error at specific points (e.g., 250 for ethyl acetate and 50 for 2-butyl alcohol). Overall, our approach exhibits greater accuracy, robustness, and consistency, with smaller fluctuations in error values across substances and conditions. These improvements highlight the versatility of our method for precise dielectric property characterization across a wide range of substances.

In this study, we conducted all the tests at a temperature of 25 25 °C. The Cole–Cole model parameters at 25 °C for the organic liquids investigated are tabulated in Table 3 [30]. These parameters encompass the relaxation time (*t*), the distribution parameter (α), and the static ($\epsilon_{s}$) and high-frequency ($\epsilon_{\infty }$) relative permittivities, corresponding to angular frequencies significantly lower and higher than the reciprocal of the relaxation time (1/t), respectively. For the purpose of experimental validation, a binary mixture composed of anhydrous ethanol and deionized water served as the analyte, with *S*-parameter measurements performed utilizing a vector network analyzer. During the course of the liquid dielectric characterization measurements, the sensor’s vertical position was maintained as constant, and a precisely measured volume of 300 μL of the analyte was introduced. Subsequent to a stabilization period to ensure thermal equilibrium, the *S*-parameters were recorded for a series of ten distinct solution mixtures, with the ethanol concentration systematically varied in 10% increments from 0% to 100%. The experimental apparatus operates at a nominal frequency of 5 GHz. A variation in the measurement frequency from 5 GHz to 4.9 GHz results in a maximum change of only 2.5% in the permittivity values for the substances enumerated in Table 3.

**Figure 16:**
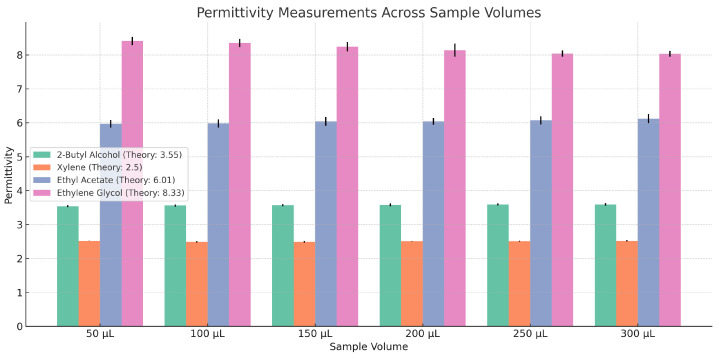
Permittivity of MUTs given the sample volumes between 50 and 300 μL.

Figure 16 presents a comparative analysis of experimentally determined dielectric constants, measured using sample volumes ranging from 50 to 300 μL, and theoretical values calculated using the Cole–Cole equation at a frequency of 5 GHz. Similar to the test in [31], we find a close agreement between measured and theoretical values across a range of materials, with dielectric constants spanning from approximately 0.15 (xylene) to 12 (methanol) at 5 GHz, which demonstrates the effectiveness of the developed measurement system in characterizing the dielectric properties of lossy liquid materials. The mean deviation between measured and theoretical values is less than 4, though a more detailed analysis shows that percentage deviations vary significantly, ranging from 0.16% to 20%, with a maximum measurement uncertainty of 2. These discrepancies are partially attributed to the intrinsic frequency dependence of the dielectric permittivity of the material under test (MUT), which introduces a source of systematic error.

The resonant frequencies predicted by the Cole–Cole dielectric relaxation model [29] for the organic liquids investigated are as follows: 3.55 GHz for 2-butyl alcohol, 2.50 GHz for xylene, 6.01 GHz for ethyl acetate, and 8.33 GHz for ethylene glycol. Experimental measurements of these resonant frequencies were conducted across sample volumes ranging from 50 μL to 300 μL, with the exception of ethylene glycol, which was measured up to 200 μL. For 2-butyl alcohol, the measured values showed minimal deviations from the theoretical prediction, ranging from 0.025 GHz to 0.039 GHz, indicating a high level of precision. Similarly, measurements for xylene demonstrated excellent agreement with the predicted value, with deviations between 0.008 GHz and 0.027 GHz, and improved accuracy at larger sample volumes. In contrast, ethyl acetate exhibited slightly greater variability, with deviations spanning from 0.097 GHz to 0.134 GHz, possibly due to its intrinsic dielectric properties. Measurements for ethylene glycol showed more significant deviations, ranging from 0.124 GHz at a sample volume of 50 μL to 0.193 GHz at 200 μL, with a noticeable trend of decreasing resonant frequency as the sample volume increased. This trend suggests that ethylene glycol’s dielectric response is highly sensitive to variations in the sample volume or experimental factors such as temperature fluctuations or subtle measurement setup inconsistencies. Compared to the free-running oscillator methodology described by [32], the approach presented here demonstrates a marked improvement in accuracy, making it particularly suitable for applications in chemical calibration and detection.

**Figure 17:**
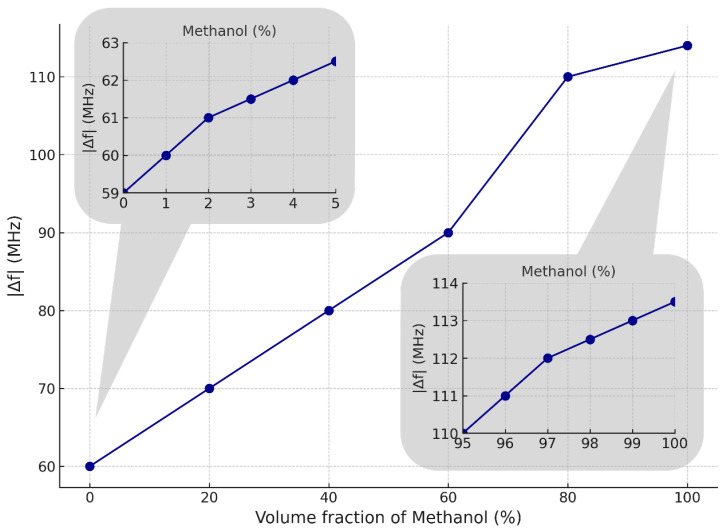
Frequency-shift measurements for ethanol–methanol mixtures.

The ability of the system to distinguish between mixtures of two organic liquids is demonstrated. For this purpose, ethanol–methanol mixtures of various volume fractions were prepared by pipetting proportional volumes of liquids that need to be mixed into a test tube and shaking them thoroughly to ensure a homogeneous solution. The frequency shift for each volume fraction of each mixture was measured five times for a sample volume of 100 μL, averaged, and plotted versus volume fraction, as shown in Figure 17. Here, a volume fraction of one liquid implies that there is one of the other. The observed frequency shift demonstrates a relatively linear relationship with increasing methanol volume fractions in an ethanol–methanol mixture. The system achieves a sensitivity of approximately 0.52 MHz of frequency shift per 1.2% increase in methanol volume fraction. Permittivity measurements, however, are highly influenced by the type and parameters of the transistors, as well as the transmission line configuration and substrate material. To mitigate these sensitivities, we can select transistors specifically optimized for high-frequency operation, ensuring low noise and stable gain, or can design the transmission line to achieve precise impedance matching and minimize parasitic effects. Certainly, we can use a substrate with homogeneous, stable dielectric properties and a low loss tangent.

## 4. Conclusions

This study presents a novel approach to dielectric constant measurement using a negative-resistance VCO integrated with a frequency synthesizer, addressing challenges associated with high-loss materials in conventional methods. By leveraging phase response variations in a planar sensor, the system achieves stable oscillations and significant frequency shifts, enabling precise characterization of lossy materials. The experimental results validate the system’s accuracy in measuring dielectric constants, demonstrating its capability to distinguish and quantify mixtures of organic liquids. Key contributions include the innovative oscillator design, robust simulation methodologies, and successful application in material characterization. The proposed system offers a compact, cost-effective solution with potential for integration into advanced microwave technologies, paving the way for enhanced permittivity measurement in diverse scientific and industrial applications. Future work will focus on optimizing harmonic suppression, extending measurement capabilities to broader frequency ranges, and refining the design for higher accuracy and miniaturization.

## Figures and Tables

**Table 1:** Notations and their value in the SIW sensor.

	Explanation	Value
μ	substrate permeability	1 (non-magnetic)
ε	substrate permittivity	2.2
*m*	number of half-cycle variations in *x*-direction	1
*n*	number of half-cycle variations in *y*-direction	1
*w*	line width of the microstrip	3.11 mm
*x*	slot width	0.2 mm
*y*	slot width	5.94 mm
$a_{1}$	ring width of the slot as well as the copper ring	2 mm
*g*	feeding line width	0.3 mm
*a*	equivalent width of SIW resonator	14.72 mm
*d*	equivalent length of SIW resonator	19.53 mm
*D*	diameter of metalized vias	1.1 mm
*p*	center-to-center distance between two consecutive vias	1.85 mm

**Table 2:** Comparison of the performance in terms of the absolute error (the smaller the better) in permittivity measurement.

	Method	50	100	150	200	250	300
2-Butyl Alcohol	[25]	0.12	0.16	0.09	0.15	0.08	0.15
	Ours	0.10	0.07	0.13	0.11	0.06	0.13
Xylene	[25]	0.11	0.19	0.15	0.22	0.09	0.14
	Ours	0.05	0.07	0.13	0.13	0.11	0.09
Ethyl Acetate	[25]	0.14	0.06	0.07	0.10	0.17	0.09
	Ours	0.12	0.08	0.13	0.15	0.08	0.11
Ethylene Glycol	[25]	0.07	0.15	0.15	0.16	0.08	0.12
	Ours	0.07	0.15	0.05	0.11	0.06	0.10

**Table 3:** Cole–Cole model parameters for liquids at 25 °C.

Organic Liquid	Relaxation *t*	Fitting α	Permittivity	
			**Angular Freq.** $\varepsilon_{s}\ll \frac{1}{t}$	**Angular Freq.** $\varepsilon_{\infty }\gg \frac{1}{t}$
Ethanol	145.28	0	24.5	4.2
Methanol	50.568	0	35.39	6.1
2-Butyl Alcohol	522	0	12.4	2.01
Xylene	9.28	0	2.39	3.27
Ethyl Acetate	3.84	0.1	7.51	2.73
Ethylene Glycol	97.4	0.1	49.6	2.9

## Data Availability

Data are contained within the article.
